# A longitudinal study on deep brain stimulation of the medial forebrain bundle for treatment-resistant depression

**DOI:** 10.1038/s41398-018-0160-4

**Published:** 2018-06-04

**Authors:** Albert J. Fenoy, Paul E. Schulz, Sudhakar Selvaraj, Christina L. Burrows, Giovanna Zunta-Soares, Kathryn Durkin, Paolo Zanotti-Fregonara, Joao Quevedo, Jair C. Soares

**Affiliations:** 1 Department of Neurosurgery, McGovern Medical School, University of Texas Health Science Center at Houston, Houston, TX USA; 2 Department of Neurology, McGovern Medical School, University of Texas Health Science Center at Houston, Houston, TX USA; 3 Center of Excellence on Mood Disorders, Department of Psychiatry and Behavioral Sciences, McGovern Medical School, University of Texas Health Science Center at Houston, Houston, TX USA; 4 Department of Neurology, Houston Methodist Research Institute and Weill Cornell Medicine, Houston, TX USA

## Abstract

Deep brain stimulation (DBS) to the superolateral branch of the medial forebrain bundle (MFB) has been reported to lead to rapid antidepressant effects. In this longitudinal study, we expand upon the initial results we reported at 26 weeks (Fenoy et al., 2016), showing sustained antidepressant effects of MFB DBS on six patients with treatment-resistant depression (TRD) over 1 year. The Montgomery-Åsberg Depression Rating Scale (MADRS) was used as the primary assessment tool. Deterministic fiber tracking was used to individually map the target area; analysis was performed to compare modulated fiber tracts between patients. Intraoperatively, upon stimulation at target, responders reported immediate increases in energy and motivation. An insertional effect was seen during the 4-week sham stimulation phase from baseline (28% mean MADRS reduction, *p* = 0.02). However, after 1 week of initiating stimulation, three of six patients had a > 50% decrease in MADRS scores relative to baseline (43% mean MADRS reduction, *p* = 0.005). One patient withdrew from study participation. At 52 weeks, four of remaining five patients have > 70% decrease in MADRS scores relative to baseline (73% mean MADRS reduction, *p* = 0.007). Evaluation of modulated fiber tracts reveals significant common orbitofrontal connectivity to the target region in all responders. Neuropsychological testing and ^18^F-fluoro-deoxyglucose-positron emission tomography cerebral metabolism evaluations performed at baseline and at 52 weeks showed minimal changes and verified safety. This longitudinal evaluation of MFB DBS demonstrated rapid antidepressant effects, as initially reported by Schlaepfer et al. (2013), and supports the use of DBS for TRD.

## Introduction

Treatment resistance is an extremely unfortunate^1,2^, not uncommon condition afflicting millions of depression patients worldwide^3,4^, leading to increased healthcare costs and overall high morbidity and mortality^5^. Various trials of deep brain stimulation (DBS) have been borne out of the need to help such patients, using historical ablative targets as a guide improved upon by the use of neuroimaging and tractography to identify possible dysfunctional circuits. Such DBS trials in treatment-resistant depression (TRD) have targeted the subcallosal cingulate gyrus (Cg25)^6–9^, ventral capsule/ventral striatum^10,11^, ventral anterior limb of the internal capsule^12^, and the nucleus accumbens (NAc)^13^, some with promising results over long-term stimulation but diminished by significant concerns of insertional effects^7^. A recent study targeting the superolateral branch of the medial forebrain bundle (MFB) reported a rapid antidepressant response in four of seven patients within 1 week increasing to six of seven patients within 4 weeks of stimulation^2^, which has been sustained over years^14^. Such rapid response is believed to be owing to the fact that the MFB lies at the center of the reward pathway connecting dopaminergic inputs from the ventral tegmental area (VTA)^15–17^ with the prefrontal cortex. We have since sought to replicate such effects, observing two of three patients at 26 weeks post stimulation onset to have > 80% improvement of depression symptoms^1^.

The goal of this longitudinal study is to elaborate upon the sustained antidepressant effects of MFB DBS on our patients with TRD over 1 year. This marks a continuation of work since our previous publication^1^ with more enrolled patients and following those initially reported upon for 1 year, and concludes our pilot study on efficacy and safety as part of an Food and Drug Administration (FDA)-approved clinical trial. Importantly, here we controlled for placebo/insertion effects after implantation, which was not planned in the Bonn study^2^. We also report on the associated neuropsychological and brain metabolic changes that occurred over the 52 weeks of this study.

## Materials and methods

This study has approval from both the University of Texas Houston Institutional Review Board (IRB) (HSC-MS-13-0004) and FDA Investigational Device Exemption (IDE) (#G130215) for the use of DBS Activa system (Medtronic, Inc., Minneapolis, MN, USA); it is registered at ClinicalTrials.gov (identifier: NCT02046330).

### Participant patients

Participants are identical to those discussed previously^1^. In brief, they were referred from local area hospitals, clinics, or ClinicalTrials.gov. Screening of candidates was performed using the Structured Clinical Interview for DSM-IV-TR (SCID-I)^18^ and all clinical records were assessed to obtain an accurate patient history.

Patients were considered eligible for the study if they met the following inclusion criteria, similar to that of other studies on DBS for TRD:^2,6–10^ (a) Major depression, severe, unipolar, diagnosed by SCID-I;^18^ (b) Hamilton Depression Rating Scale (HDRS_29_)^19^ score > 21 on the first set of items; (c) MADRS^20^ score > 21; (d) Global Assessment of Function^21^ score of < 45; (e) a recurrent ( ≥ 4 episodes) or chronic (episode duration ≥ 2 y) course and a minimum of 5 y since the onset of the first depressive episode; (f) age 22–65 y; (g) refractory to > 6 weeks of multiple medication regimens; (h) refractory to > 20 sessions psychotherapy; (i) refractory to a trial of electroconvulsive therapy ( ≥ 6 bilateral treatments). Exclusion criteria were the following: (a) current or past bipolar disorder, non-affective psychotic disorder, schizophrenia, or schizoaffective disorder; (b) severe personality disorder as assessed by SCID-II^22^ and Millon Clinical Multiaxial Inventory-III (MCMI-III);^23^ (c) significant neurological disorder; (d) previous surgery to destroy the target region of the brain; (e) surgical contraindications to DBS.

Recruitment for the subjects described herein began in October 2014 and concluded in June 2016; the inclusion of Patient 6 was in August 2016. Owing to a change in our IRB agreement, the latter patients in our study required insurance authorization prior to enrollment, which delayed quick inclusion.

### Study protocol

**Fig. 1:**
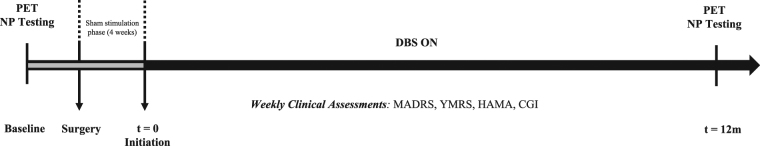
Study protocol. DBS, deep brain stimulation; SE, side effects; PET, positron emission tomography; Hz, Hertz; us, microseconds; V, volts; MADRS, Montgomery-Åsburg Depression Rating Scale, HAM-A, Hamilton Anxiety Scale; YMRS, Young Mania Rating Scale; CGI, Clinical Global Impressions

Informed consent was obtained from all subjects prior to participation. Patients were required to maintain their same medication for 6 weeks before and 6 months after surgery. Six patients were enrolled into this study; five completed the 52-week protocol, with one patient dropping out after 6 weeks. Psychiatric assessments were performed on a weekly basis by a psychiatrist independent of the programmer starting one week following implantation. All assessments were performed in person at the University of Texas, Houston Behavioral and Biomedical Sciences Building; Patient 2 suddenly withdrew from the study to attend to family circumstances 3000 miles away. For the initial 4 weeks following surgery, the patients entered a single-blind sham stimulation phase. At the conclusion of this period, they were unblinded and stimulation initiated (Fig. 1). At baseline and repeated at 12 months, cognitive functioning was assessed in study patients by a standardized comprehensive neuropsychological test battery (see Supplementary Table 1).

The primary outcome measure was the antidepressant response on the Montgomery-Åsberg Depression Rating Scale (MADRS), with a 50% reduction of depressive symptom severity being interpreted as a positive response. Secondary outcome measures included the Hamilton Anxiety Scale (HAM-A)^24^, the Young Mania Rating Scale (YMRS)^25^, and the Clinical Global Impressions (CGI)^26^. Each was collected during the weekly assessment; the 29 point HDRS_29_^19^ was completed at baseline and at 6 and 12 months and the GAF^21^ was completed at baseline and at 12 months.

Safety information and adverse events regarding the treatment method were recorded in a standardized document according to FDA regulations. Safety testing included neuropsychological evaluation to rule out cognitive effects of DBS.

### Imaging and targeting protocol

Pre-operatively, diffusion, T2, and T1-weighted post-gadolinium sequences were acquired on a 3-Tesla magnetic resonance imaging (MRI) system, as described previously^1^.

Each patient’s MFB was individually mapped by means of deterministic fiber tracking using diffusion sequences. An area lateral to the VTA, anterior to the red nucleus, and posterior to the mammillary bodies was used as the seed region, as described by Coenen et al.^27^. Using StealthViz software (Medtronic, Inc.), such mapping resulted in clear projections of the slMFB through to the medial prefrontal cortex. Results of such fiber tracking were then transferred to the stereotactic planning software (Framelink, Medtronic, Inc.), where the center of this fiber bundle superolateral to the VTA was used as the target for the DBS electrode, as previously described^1,2^.

### Surgical procedure and electrode positions

DBS surgery was performed using the Leksell frame (Elekta, Sweden) and following our standard protocol^28,29^, with the patient awake and predominantly under local anesthesia by use of mild sedation with propofol (Diprivan, AstraZeneca, London) during burr hole placement. Two or three microelectrodes (FHC, Bowdoin, ME) were descended down to target simultaneously and recording performed. Commencement with a right or left sided brain trajectory was alternated.

Intraoperative test stimulation was then performed at the target point through a chosen microelectrode (continuous 5 min of monopolar macrostimulation through the microelectrode cannula: 125 Hz, 75 μs, 2–3 mA) to test for acute mood and behavioral changes as well as to identify the threshold of oculomotor side effects (diplopia). As described previously^1,2^, an increase in “appetitive motivation” (visual contact, friendly conversation, feelings of energy) and mood improvement typically occurred following proper electrode positioning; if no such effects were observed or if threshold of side effects (diplopia) was too low, a change in microelectrode test position was made (still within the tractography-defined seed region). Self-report ratings were obtained on the Positive and Negative Affect Scale (PANAS)^30^ at baseline and repeated at the culmination of a 5-min stimulation session; major increases in positive totals and decreases in negative totals were seen in most patients and predicted proper target location and/or future response, as previously described (Supplemental Fig. 1). The Medtronic 3389 electrode was then placed and secured without further testing. Importantly, intraoperative effects of appetitive motivation experienced during stimulation at proper target location on the first side were not seen to carry over when testing the contralateral side; patients returned to their baseline levels as assessed by repeat PANAS and by subjective report. For each side, intraoperative 3D imaging was then performed (O-Arm Surgical Imaging System (Medtronic, Inc.)) to detect deviations of electrode from target trajectory, prior to removal of frame and then placement of lead extensions and Activa PC pulse generator.

### Post-operative imaging analysis

Using a deterministic tractography algorithm (StealthViz), all pathways intersecting a volume of tissue activation (VTA) were modeled by a spherical region around the therapeutic electrode contacts. This was accomplished by first localizing the cathodal contact center on a thin slice (1 mm) CT volume following co-registration to the pre-surgical T2 anatomical volume. The radius of the VTA was estimated using therapeutic cathodal electrode contact parameters and an isotropic model as proposed by Butson et al.^31^, which was then used as the seed region of interest. The fibers passing through this region give an estimate of modulated fiber tracts that could be compared with the pre-operatively targeted tracts.

### PET protocol

A baseline ^18^F-fluoro-deoxyglucose (FDG)-PET measurement of regional cerebral glucose metabolism was obtained during the week before surgery on each patient and was repeated at 12 months post surgery. One patient had only the baseline study, and was therefore excluded from the PET analysis. FDG-PET scans were performed on a 3D PET/CT scanner (General Electric (GE) Discovery ST) at Memorial Hermann Hospital, Houston. All patients were fasting and were asked to refrain from coffee and alcohol for a period of 4 h before each session. All scans were performed between 9 AM and noon. Each scan started ~ 30 min after intravenous injection of 11.3 ± 2.4 mCi of ^18^F-FDG (range, 8.6–15 mCi). A low-dose CT for attenuation correction was first performed. Then, emission data were acquired during a 35-min period, with a voxel size of 2.34 × 2.34 × 3.27 mm. The preprocessing of the brain images (such as the segmentation of the MRI and the co-registration of the PET to the MRI) and the calculation of the uptake in the regions of interest was done with the Pneuro pipeline implemented in Pmod 3.9 (Pmod technologies, Zurich, Switzerland), using the Hammers atlas^32^. Glucose metabolism was studied in seven preselected regions (frontal lobes, cingulate region, caudate, putamen, substantia nigra, nucleus accumbens, and hippocampus) on the basis of prior studies^2,8^. The frontal lobe included all subregions of the frontal lobe, except the precentral gyrus, and the cingulate region included the anterior and posterior cingulum, as well as the subgenual cortex. Each region was the average of the left and right region, and its uptake was normalized over that of the cerebellum. The uptake in the PET regions was compared with paired *t* tests with Bonferroni correction for multiple comparisons.

### Stimulation onset and parameter adjustments

After implantation, each patient was single-blinded to stimulation onset for a 4-week period. Weekly assessments occurred between the patient and psychiatry team. During this 4-week sham stimulation phase, each contact was individually explored to assess for threshold of side effects (oculomotor effects, dizziness). Such contact assessment/programming sessions were limited to 5 min and were useful in promoting patients’ “blindedness” regarding their stimulation status, as each patient participated with the contact exploration; all patients were uniformly turned off at the end of each session. By 4 weeks post stimulation, each patient was unblinded to stimulation status and all received open-label active stimulation.

Choice of configuration and parameters occurred during the sham stimulation phase once post-operative deterministic tractography was performed, using the actual electrode location to decide, which contact(s) would best modulate the target fibers for a specified VTA, which from our previous report^1^ seemed to have extensive orbitofrontal cortex (OFC) projections in responder patients. In most cases, comparison of the estimated modulated fiber tracts with the pre-operatively targeted tracts showed largely overlapping territories. Based on this analysis, the initial electrode configuration and parameter choice for most patients was identical to that used by Schlaepfer et al.:^2^ 1 + 2–3-, 130 Hz, 60 µs, 3 V. Constant voltage stimulation was applied. Oculomotor side effects, if observed, led to a smaller starting amplitude; these side effects abated with parameter change. Adjustments in parameters (amplitude and pulse width, not frequency) and/or configuration (with contacts still within the target area tractographic map) were made for some patients over the course of the study if there was an increase in MADRS scores or lack of antidepressant response for 2 or more consecutive weeks. PANAS self-report ratings were completed during each programming change.

Medication was kept constant to the degree possible in all patients. For Patient 1 and Patient 3, antidepressant medication changes were made after 6 weeks and were reported to the IRB and FDA. There were no adjunctive psychotherapy treatment measures administered to any patient.

### Statistical analysis

Weekly clinical data (MADRS, HAM-A, YMRS, CGI) were analyzed with descriptive methods (mean, standard deviation, frequency). Paired Student’s *t* test was used to compare data across different time points. FDR (false discovery rate) correction (neuropsychological data) and Bonferroni correction (PET data) were applied for multiple comparisons.

## Results

All six patients were diagnosed with severe, treatment-resistant depression, having multiple major depressive episodes and a mean length of current major depressive episode of 5.7 years (SD = 2.1 y). At the time of implantation, the mean number of antidepressant medications was 2.5 (SD = 1.0) with mean lifetime use of 14.8 (SD = 5.9). All patients had received electroconvulsive therapy and psychotherapy without lasting or sufficient treatment response (Table 1).

**Table 1:** Patient demographic data and clinical results

Variable	Patient 1	Patient 2	Patient 3	Patient 4	Patient 5	Patient 6	Mean (SD)
Age at implant (y)	55	47	49	34	51	65	50.2 (10.2)
Sex	F	F	M	M	F	F	
SCID-I primary diagnosis	MDD	MDD	MDD	MDD	MDD	MDD	
SCID-I secondary diagnosis	PTSD, social phobia, adjustment disorder with anxiety	Specific phobia, anorexia nervosa, past opiate dependence			Past history of bulimia nervosa	Past history of alcohol abuse	
SCID-II (personality disorders (PD) and traits)	Depressive PD, dependent PD with avoidant features	Depressive PD, antisocial and obsessive compulsive traits	Depressive PD with schizoid, avoidant features; obsessive compulsive PD	Depressive, negativistic, and obsessive compulsive PD	Obsessive compulsive PD	–	
SCID-III medical comorbidities	Hypertension, hypothyroidism, hyperlipidemia, migraines, type II diabetes	Hysterectomy	Hypertension	Irritable bowel syndrome, hypertension	Type I diabetes, hypertension, hyperlipidemia, rheumatoid & osteoarthritis	History of ovarian cancer, hysterectomy, splenectomy, hyperlipidemia	
Education (y)	20	13	13	16	17	15	15.7 (2.7)
Working status	Unable due to MDD	On disability	On FMLA at screening	Full-time	Unable due to MDD	Retired	
Previous depressive episodes (#)	30	5	2	10	60	200	51.2 (76.1)
Duration (y) of current episode	4	6	9	6	6	3	5.7 (2.1)
Age at Onset (y)	17	15	21	13	21	4	15.2 (6.3)
Hospitalizations (#)	10	3	0	0	0	0	2.2 (4.0)
Antidepressants at implant (#)	4	2	2	3	3	1	2.5 (1.0)
Antidepressants tried (lifetime#)	18	18	22	12	14	5	14.8 (5.9)
Psychotherapy (# y in therapy)	20	4	1	5	10	4	7.3 (6.9)
Past ECT (# of times)	12^a^	6	6^a^	30^a^	14^a^	12^a^	13.3 (8.8)
Suicide attempts (#)	5	1	0	0	0	0	1.0 (2.0)
Scores (longitudinal)							
MADRS at inclusion	35	37	34	30	37	37	35.0 (2.8)
MADRS – 1 w^b^	12	18	29	7	29	25	20.0 (9.2)
MADRS – 26 w	6	-	30	4	9	2	10.2 (11.4)
MADRS – 52 w	6	-	27	1	11	0	9.0 (10.9)
No. of months with response^c^/total	9/12	1/1	0/12	12/12	7/12	12/12	
HDRS-29 at inclusion	42	39	37	41	39	39	39.5 (1.8)
HDRS-29 – 26 w	19	-	28	3	6	10	13.2 (10.2)
HDRS-29 – 52 w	3	-	31	2	6	0	8.4 (12.8)
CGI at inclusion	6	5	5	5	5	5	5.2 (0.4)
CGI – 1 w	2	4	5	2	5	5	3.8 (1.5)
CGI – 26 w	2	-	4	2	2	1	2.2 (1.1)
CGI – 52 w	2	-	5	1	2	1	2.2 (1.6)
HAM-A at inclusion	14	24	10	20	26	18	18.7 (6.0)
HAM-A – 1 w	7	13	15	7	17	13	12.0 (4.1)
HAM-A – 26 w	4	-	13	4	4	3	5.6 (4.2)
HAM-A – 52 w	2	-	13	3	5	1	4.8 (4.8)
YMRS at inclusion	1	4	2	2	2	0	1.8 (1.3)
YMRS – 1 w	0	3	0	3	1	0	1.2 (1.5)
YMRS – 26 w	0	-	2	0	0	0	0.4 (0.9)
YMRS – 52 w	0	-	2	1	1	0	0.8 (0.8)

^a^With transient improvement

^b^w = weeks post stimulation onset

^c^Response = > 50% improvement from baseline MADRS score

SCID, Structured Clinical Interview for DSM-IV- TR; MDD, major depressive disorder; PTSD, post-traumatic stress disorder; ECT, electroconvulsive therapy; DBS, deep brain stimulation; MADRS, Montgomery-Åsburg Depression Rating Scale; HDRS, Hamilton Depression Scale; HAM-A, Hamilton Anxiety Scale; YMRS, Young Mania Rating Scale; CGI, Clinical Global Impressions; SD, standard deviation; PD, personality disorder; FMLA, Family Medical Leave Act

### Clinical outcomes

**Fig. 2:**
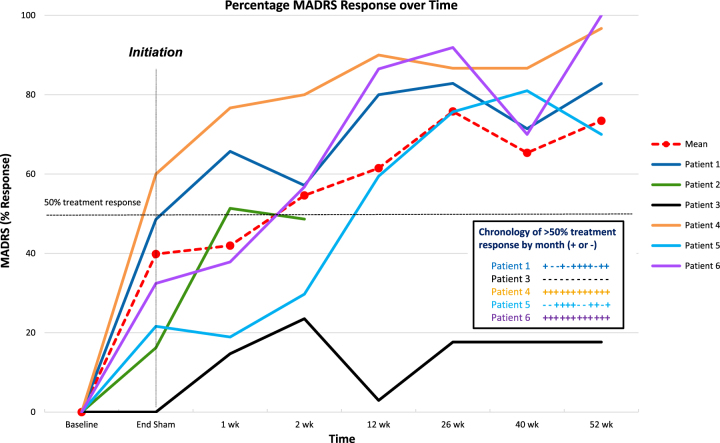
MADRS scores recorded over time. Initiation = bilateral DBS ON at *t* = 4w OFF following MADRS assessment. End-sham = 4w OFF. DBS initiation begun after End-Sham assessment. Mean % change MADRS baseline–12 w ON = 64%; mean % change MADRS baseline–52 w = 73%. MADRS, Montgomery-Åsburg Depression Rating Scale. Insert: Chronology of > 50% treatment response by month ( ±) for each patient for 12 months

Intraoperative test stimulation at target incurred in each responder increased vigilance, eye contact, and engagement in conversation, as well as self-reported feelings of energy and motivation concordant with mood improvement, similar to that described by Schlaepfer et al.^2^. Patient 3 did not experience any effects from intraoperative stimulation aside from oculomotor effects (diplopia). All patients were uniformly OFF during the initial 4 weeks post surgery (sham stimulation phase). During this phase, there was a significant mean change in mood, where the mean difference in MADRS score between baseline and the end of sham stimulation was 10 points (28% reduction, *p* = 0.02). See Fig. 2. After initiating stimulation, 3 of 6 patients had a > 50% decrease in MADRS scores relative to baseline at 7 days. The difference in MADRS score over the 1 week between the end of sham stimulation and the first active stimulation assessment was significant (mean change = 5 points, 20% reduction, *p* = 0.05) as was the difference between baseline and 1 week of active stimulation (mean change = 15 points, 43% reduction, *p* = 0.005). The difference in MADRS score between baseline and week 2 of active stimulation was further significant (mean change = 17 points, 49% reduction, *p* = 0.001), at which time point four of six patients had ≥ 50% decrease in MADRS scores relative to baseline. As discussed previously, one of these patients (Patient 2) withdrew from study participation after 2 weeks after stimulation onset for family reasons.

At 12 weeks post stimulation onset, four of the five remaining patients had ≥ 60% reduction in MADRS scores from baseline and were classified as responders (Fig. 2). There was fluctuation in response seen over time. Patients 1 and 5 had acute exacerbations of their depression-related to stressors in their lives (family related), leading to periodic worsening of symptoms. During successive weeks of worsening mood, stimulation increases were performed in both patients, with medication changes made to Patient 1. Ultimately, all four responder patients had > 50% of their evaluations meeting response criteria of ≥ 50% reduction in MADRS scores from baseline (Fig. 2). At 52 weeks, these same patients continued to have > 70% reduction in MADRS scores from baseline (73% mean MADRS reduction, *p* = 0.007). These four responder patients meet criteria for remission with HAM-D scores < 7 at 52 weeks. No negative neuropsychological effects were demonstrated, with most scores stable compared with baseline for the five patients (see Supplementary Table 1).

YMRS, CGI, HAM-A results for baseline and after 1, 26, and 52 weeks of stimulation are shown in Table 1. The group mean interval difference was not significantly different from baseline in YMRS. CGI scores were significantly reduced in all patients from a mean score of 5.2 (SD = 0.4) to 2.2 (SD = 1.6) over 52 weeks, mean change = 3 points (*p* = 0.02). If only the four responders are considered, there was a mean change of 3.7 points at 52 weeks (final mean score 1.5, SD = 0.6, *p* < 0.001). Overall mean scores were significantly reduced in the HAM-A, from a mean score of 18.7 (SD = 6.0) to 4.8 (SD = 4.8) over 52 weeks, mean change = 13.9 points (*p* = 0.04). However, regarding the four responders, scores in the HAM-A showed a significant 49% reduction from baseline (mean = 19.5 points, SD = 5) at week 1 (mean = 10, SD = 3.6, *p* = 0.01), increasing to an 85% reduction from baseline at 52 weeks (mean = 2.8 points, SD = 1.7, *p* = 0.003).

### Contact location and parameters

**Fig. 3:**
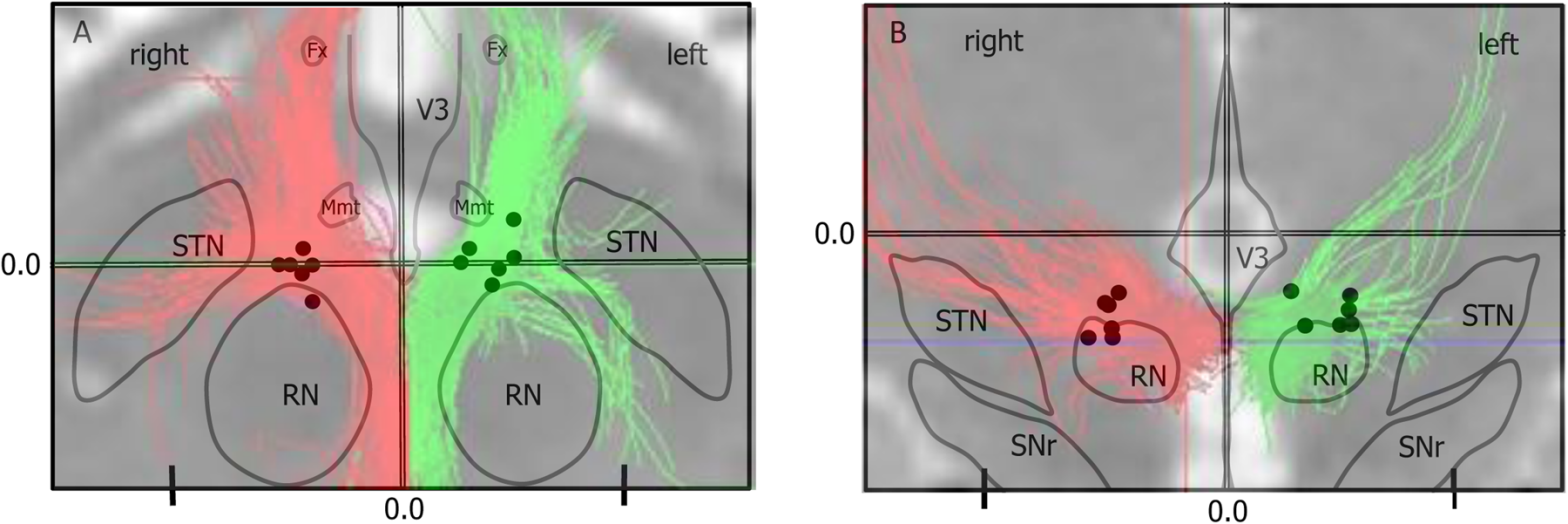
Representation of active cathodal contacts in two planes for each of the six patients presented in this series, superimposed upon the deterministic tractography-defined target of the medial forebrain bundle for Patient 1; this is presented on adaptations of stereotactic atlas slices from Schaltenbrand and Wahren^50^. **a** H.v. 4.5, axial view **b** F.p. 3.0, Coronal view. STN = subthalamic nucleus; RN = red nucleus; SNr = substantia nigra; Mmt = mammillothalamic tract; Fx = fornix; V3 = 3rd ventricle

For the five patients that completed the study, at 52 weeks the mean amplitude of stimulation was 3.8 V (SD = 1.2) and the mean pulse width was 64 µs (SD = 8.4); frequency was uniformly kept at 130 Hz in all patients. Patient 2 departed the study with parameters 130 Hz, 60 µs, 3 V. Please refer to Fig. 3, depicting locations of the six active cathodal contacts.*-

### Adverse events

The most common adverse event was diplopia, usually vertical, seen in all patients upon larger parameter changes and with specific contact use, which was transient and remitted upon selection to a different setting. There were no incidences of post-operative hemorrhage or infection; headache was transient in some patients.

### Connectivity patterns

**Fig. 4:**
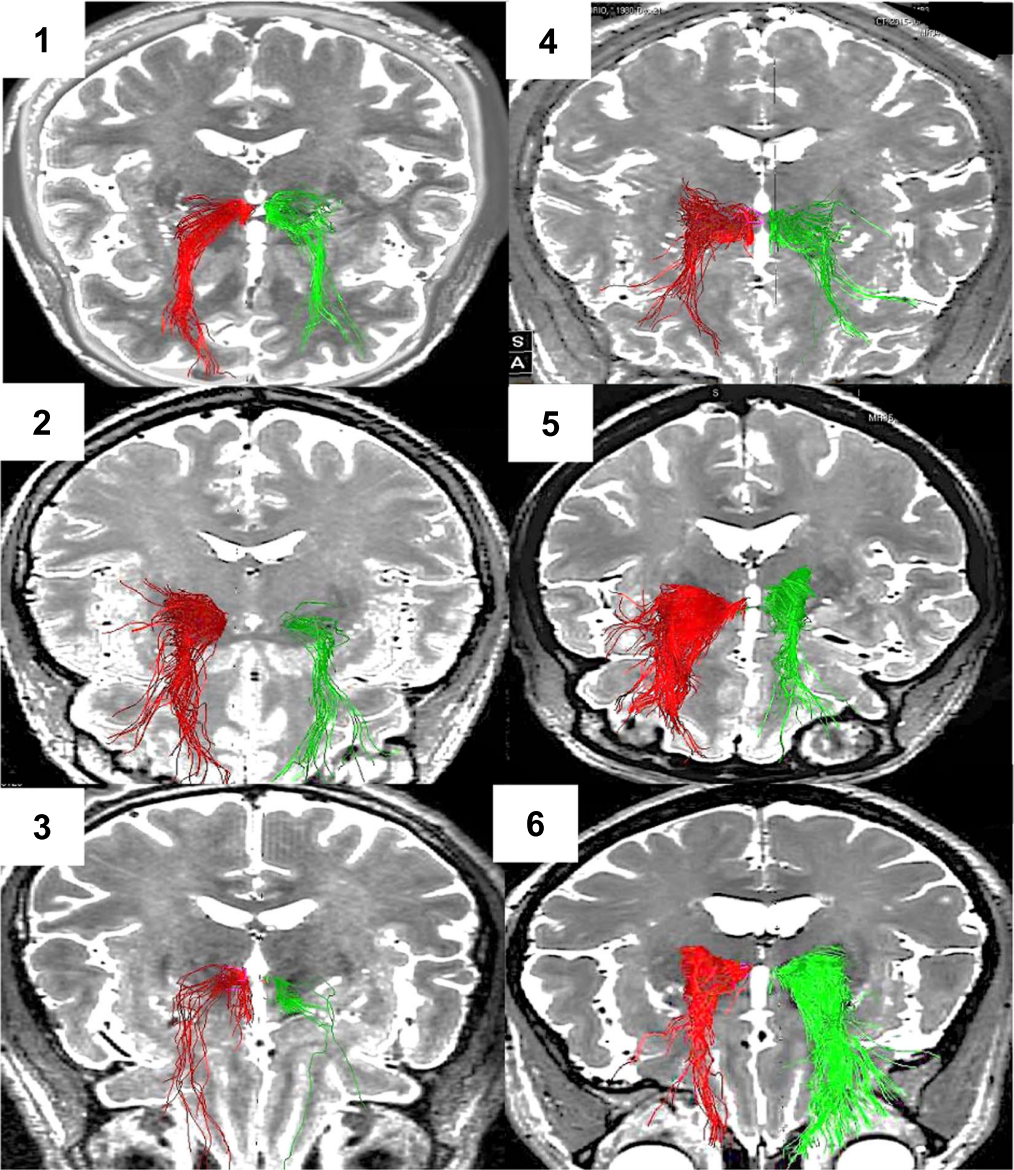
Depiction of modulated fiber tracts (assuming an isotropic model) from active cathodal contacts in patients #1–#6. Chronological order of implantation. Significant orbitofrontal connectivity to the MFB target region seen in all responder patients but minimally seen in the non-responder Patient no. 3

Each patient’s individual deterministic diffusion tensor imaging was used to define their individual target site (Fig. 3). After implantation, we used the post-op CT-defined location of the active electrode contact to estimate modulated fiber tracts (see Methods). All but the non-responder have strong connectivity between the target location of the active cathodal contact and the OFC, similar to their individual planning images. The non-responder patient has limited, sparse connectivity between the seed region and the prefrontal cortex on both targeting images and upon modeling (Fig. 4).

### PET results

PET data did not show significant changes in glucose metabolism 12 months after DBS, compared with baseline. There was a significant post-treatment reduction of the ^18^FDG uptake in the caudate (*p* = 0.027), but it did not survive correction for multiple comparisons. No other regions showed a significant change.

## Discussion

These longitudinal results support our preliminary ones^1^ in demonstrating that MFB DBS incurs a rapid antidepressant effect, as evidenced in three patients after just 1 week of stimulation. By 26 weeks and continued through 1 year, four of the five patients completing this study have a sustained > 70% reduction of depression scores from baseline. This 80% response rate is significant, and gives credence to the efficacy of MFB DBS and to the rapidity of response. These results confirm the observations of Schlaepfer et al.^2^.

In this study, patients were single-blinded to stimulation onset to control for possible insertional effects that might occur after implantation in the DBS-OFF condition, which was not evaluated by Schlaepfer et al.^2^ There was a significant (*p* = 0.02) mean decrease in MADRS scores between baseline and end-sham stimulation, however, this was not seen in all patients. Such an “insertion effect” was seen to be significant in other DBS studies with a similar 4-week sham stimulation period^7^, possibly owing to acute inflammatory mediators^33^, or glial released neurotransmitters^34,35^. It is possible that whatever neurochemical change that occurs from insertion and which induces mood improvement may take longer than 4 weeks to subside. Interestingly, although such “insertion effects” may provide a confound, after only 1 week of active stimulation there was again a significant decrease in MADRS score from end of sham (mean change = 5 points, *p* = 0.05) with a more marked difference from baseline (mean change = 15 points, 43% reduction, *p* = 0.005; Fig. 2). A longer sham stimulation phase would better separate any insertion effect from treatment effect. However, it is highly unlikely that the response rate seen at 52 weeks is attributed to placebo.

Observed and self-reported intraoperative effects (i.e., motivational comments, alertness, energy level) as well as changes in PANAS ratings were striking after 5 min of stimulation at target and were used to help choose trajectory. Such effects were seen upon unilateral right or left MFB stimulation and correlated well with ultimate response.

### Connectivity analysis

As before, we used individual deterministic fiber tracking of each patient’s diffusion tensor sequence to individually map the target of the MFB, as described^1,27^, and then evaluated the modulated fibers from an estimated VTA model. In the responder patients, there was significant fiber tract modulation that closely approximated the targeted fibers (Fig. 4), with extensive connectivity to the OFC and Brodmann Area (BA) 10. There appears to be common involvement of the forceps minor among other white matter tracts connecting the target region to the OFC, as seen in other DBS studies in TRD involving other targeted locations^36^. It is possible that such modulation of specific cortical structures, including BA 10, and involvement of certain white matter tracts is a commonality universally required for antidepressant effect. In the non-responder (Patient 3), both the targeted and modulated fibers lack extensive connectivity to the OFC, which could be an underlying contributor for the lack of response.

### Mechanisms of efficacy

The MFB is a key structure of the mesolimbic-dopamine reward system^37^, connecting the VTA with the lateral hypothalamus and the NAc, which then connects to the limbic PFC^38,39^. The VTA is an important relay station in this dopaminergic reward circuit^40–44^. Optogenetic studies have confirmed that dopaminergic cell firing from the VTA can regulate depressive behavior^45^ and that inhibition of the VTA-mPFC projection promotes depression susceptibility^46^. It is believed that modulation of the MFB via DBS may recruit the descending glutamatergic fibers from the mPFC to the VTA and may indirectly regulate VTA dopaminergic firing^47^ as well as modulate upstream cortical regions^2^. Although such fiber tracts have been identified in humans using dTi^27^, there has been no functional imaging analysis to verify modulation-induced reward network change and remains speculative.

Notably, we did not find any statistically significant changes (corrected for multiple comparisons) in FDG uptake after DBS, as measured by PET. This could be explained by the low number of subjects or by the fact that DBS did not affect the metabolism of glucose. It should also be noted that a lack of correlation between clinical and PET outcome measures is not uncommon in depression studies^48,49^.

### Limitations

This is a longitudinal report on six patients at 1 year post stimulation, and marks the conclusion of our pilot study on efficacy and safety. As one patient withdrew from the study, only five patients completed 1 year and provide longitudinal observational data. Owing to a small sample size, limited conclusions can be drawn from this data set to generalize its overall efficacy. Nonetheless, four of five patients were responders at 12 weeks post stimulation onset. Fluctuation of response was seen in some patients throughout the time course of the study, but at 1 year all responders met remission criteria without adverse effects, which is very encouraging.

## Conclusion

This report concludes our pilot study of our FDA-approved clinical trial on the safety and efficacy of MFB DBS in TRD, and has provided very encouraging results. Four out of five patients had > 70% decrease in MADRS scores within 12 weeks of stimulation onset, where three of these patients met response criteria within 1 week of stimulation. There was also significant improvement in HAM-A and CGI, which suggest reduced severity of overall symptoms. The fact that these effects occur early but are sustained through 1 year argue against placebo, but the “insertional effect” before stimulation cannot be ignored. Structural connectivity analysis has elucidated that there seems to be essential, common tracts that are modulated in all responder patients, especially connectivity to OFC from the midbrain. We will use these data as a basis to devise further studies with increased enrollment to confirm such rapid efficacy, to better define the insertional effect, and to confirm the cortical areas involved in treatment response.

## Electronic supplementary material

Supplementary Table 1: Neuropsychological Results at Baseline and 1 Year Follow-up with the Significance of the Mean Change

Supplemental table legend
