# Supplementary material for: A longitudinal study on deep brain stimulation of the medial forebrain bundle for treatment-resistant depression
Source: Transl Psychiatry. 2018 Jun 4;8:111. doi: 10.1038/s41398-018-0160-4 (PMC5986795; doi:10.1038/s41398-018-0160-4)
Supplement: Supplementary file 1 — Supplementary Table 1: Neuropsychological Results at Baseline and 1 Year Follow-up with the Significance of the Mean Change [file 41398_2018_160_MOESM1_ESM.docx]

**Supplementary Table 1: Neuropsychological Results at Baseline and 1 Year—Significance of the Mean Change.**

|  |  | **Baseline** | | **1 year** | | **Change** | |  |  |  |  |
| --- | --- | --- | --- | --- | --- | --- | --- | --- | --- | --- | --- |
| Cognitive Domain | *n* | *M* | *SD* | *M* | *SD* | *M* | *SD* | *t* | *df* | *p* | Ref. |
| General Cognitive |  |  |  |  |  |  |  |  |  |  |  |
| TOPF | 5 | 0.59 | 0.47 | 0.29 | 0.44 | -0.20 | 0.47 | -0.74 | 2 | 0.85 | 49 |
| MoCA (Raw) | 5 | 26.00 | 1.22 | 26.20 | 1.92 | 0.20 | 1.30 | 0.34 | 4 | 0.97 | 50 |
| Learning and Memory |  |  |  |  |  |  |  |  |  |  |  |
| CVLT-II Total Trials 1-5 | 5 | 0.14 | 0.76 | -0.26 | 1.09 | -0.40 | 1.80 | -0.50 | 4 | 0.87 | 51 |
| CVLT-II LDFR | 5 | -1.10 | 1.47 | -1.30 | 1.64 | -0.20 | 2.89 | -0.15 | 4 | 0.97 | 51 |
| CVLT-II Recognition | 5 | -1.00 | 0.50 | -1.00 | 0.94 | 0.00 | 0.71 | 0.00 | 4 | 1.00 | 51 |
| BLT Total Trials 1-5 | 5 | -1.21 | 0.74 | -0.58 | 1.00 | 0.63 | 1.04 | 1.35 | 4 | 0.75 | 52 |
| BLT LDFR | 5 | -0.89 | 0.64 | -1.20 | 0.90 | -0.31 | 0.80 | -0.87 | 4 | 0.78 | 52 |
| BLT Recognition | 5 | -1.27 | 1.10 | -1.00 | 0.75 | 0.27 | 1.15 | 0.53 | 4 | 0.87 | 52 |
| Attention/Processing Speed |  |  |  |  |  |  |  |  |  |  |  |
| WAIS-IV Digit Span | 5 | 0.07 | 0.76 | -0.33 | 1.00 | -0.40 | 0.43 | -2.06 | 4 | 0.75 | 53 |
| TMT A | 5 | 0.08 | 0.66 | -0.74 | 1.00 | -0.82 | 1.04 | -1.75 | 4 | 0.75 | 54,55 |
| Stroop Word Reading | 5 | -1.64 | 1.00 | -1.38 | 0.94 | 0.26 | 0.64 | 0.90 | 4 | 0.78 | 56 |
| Stroop Color Naming | 5 | -1.40 | 1.04 | -1.44 | 1.47 | -0.04 | 0.78 | -0.12 | 4 | 0.97 | 56 |
| Language |  |  |  |  |  |  |  |  |  |  |  |
| BNT | 5 | -0.86 | 1.30 | -0.74 | 1.19 | 0.12 | 0.50 | 0.54 | 4 | 0.87 | 54,57 |
| COWA | 5 | -0.93 | 0.38 | -1.26 | 0.50 | -0.45 | 0.54 | -1.65 | 3 | 0.75 | 54,58 |
| Animal Fluency | 5 | -0.65 | 0.77 | -1.76 | 1.23 | -1.03 | 1.24 | -1.65 | 3 | 0.75 | 54,58 |
| Motor |  |  |  |  |  |  |  |  |  |  |  |
| GPT (dom hand) | 5 | -1.38 | 0.55 | -1.84 | 1.28 | -0.46 | 1.08 | -0.95 | 4 | 0.78 | 54,59 |
| GPT (non-dom hand) | 5 | -1.36 | 0.46 | -1.70 | 1.48 | -0.34 | 1.43 | -0.53 | 4 | 0.87 | 54,59 |
| Visual-spatial |  |  |  |  |  |  |  |  |  |  |  |
| JLO | 5 | -0.14 | 3.59 | 0.11 | 3.26 | 0.25 | 3.29 | 0.17 | 4 | 0.97 | 60,61 |
| Hooper VOT | 5 | -0.36 | 0.82 | -0.36 | 0.88 | 0.00 | 0.60 | -0.00 | 4 | 1.00 | 62 |
| Executive Functions |  |  |  |  |  |  |  |  |  |  |  |
| TMT B | 5 | -0.38 | 0.70 | -1.22 | 1.90 | -0.84 | 1.55 | -1.21 | 4 | 0.78 | 54,55 |
| WASI-II Similarities | 5 | -0.04 | 0.25 | 0.50 | 0.87 | 0.54 | 0.90 | 1.35 | 4 | 0.75 | 63 |
| WASI-II Matrix Reasoning | 5 | 0.32 | 0.56 | 0.26 | 0.93 | -0.06 | 0.89 | -0.15 | 4 | 0.97 | 63 |
| Stroop Inhibition | 5 | -0.40 | 1.05 | -0.60 | 1.02 | -0.20 | 0.41 | -1.10 | 4 | 0.78 | 56 |
| DKEFS Sorting CCS | 5 | 0.33 | 0.53 | 0.67 | 1.43 | 0.33 | 1.05 | 0.71 | 4 | 0.85 | 64 |
| DKEFS Tower Test | 5 | -0.07 | 0.86 | 0.27 | 0.95 | 0.33 | 0.82 | 0.91 | 4 | 0.78 | 64 |
| Ruff TUD | 5 | -0.52 | 0.73 | -1.20 | 0.88 | -0.68 | 0.34 | -4.51 | 4 | 0.53 | 65 |
| Ruff TPE | 5 | -0.25 | 1.18 | -0.29 | 0.43 | -0.04 | 1.22 | -0.07 | 4 | 0.98 | 65 |
| Ruff ER | 5 | -0.26 | 1.37 | 0.70 | 0.86 | 0.96 | 1.61 | 1.33 | 4 | 0.75 | 65 |
| IGT Net Total | 5 | -0.50 | 0.48 | -0.88 | 0.87 | -0.38 | 0.82 | -1.04 | 4 | 0.78 | 66 |
| FrSBe Apathy | 5 | 4.62 | 2.04 | 2.76 | 2.63 | -1.86 | 1.50 | -2.77 | 4 | 0.64 | 67 |
| FrSBe Disinhibition | 5 | -0.10 | 1.16 | -0.34 | 0.98 | -0.24 | 1.07 | -0.50 | 4 | 0.87 | 67 |
| FrSBe Executive Dysfunction | 5 | 2.16 | 0.98 | 0.90 | 1.83 | -1.26 | 1.07 | -2.64 | 4 | 0.64 | 67 |
| FrSBe Total | 5 | 2.64 | 1.24 | 1.26 | 1.84 | -1.38 | 1.29 | -2.39 | 4 | 0.64 | 67 |
| Note: M = mean Z score, SD = standard deviation, df = degrees of freedom; Ref. = Reference #; p values were corrected by FDR (false discovery rate). | | | | | | | | | | | |

**REFERENCES**

1. Wechsler D. *Test of Premorbid Functioning*. The Psychological Corporation: San Antonio, TX, 2009.
2. Nasreddine ZS, Phillips NS, Bédirian V, Charbonneau S, Whitehead V, Collin I et al. The Montreal Cognitive Assessment, MoCA: a brief screening tool for mild cognitive impairment.  J Am Geriatr Soc 2005; *53*(4):695-699.
3. Delis DC, Kramer JH, Kaplan E, Ober BA. *California Verbal Learning Test-Second Edition.*The Psychological Corporation: San Antonio, TX, 2000.
4. Brown FC, Roth RM, Saykin AJ, Gibson-Beverly, G. A new measure of visual location learning and memory: development and psychometric properties for the Brown Location Test (BLT).  Clin Neuropsychol 2007; 21:811–825.
5. Wechsler D. *Wechsler Adult Intelligence Scale–Fourth Edition*. Pearson: San Antonio, TX, 2008.
6. Heaton RK, Miller SW, Taylor MJ, Grant I. *Revised comprehensive norms for an expanded Halstead Reitan battery: demographically adjusted neuropsychological norms for African Americans and Caucasian adults.* PAR: Lutz, FL, 2004.
7. Reitan RM. The relation of the Trail Making Test to organic brain damage. J Consult Psychol 1955*; 19:*393-394.
8. Golden CJ, Freshwater SM. *Stroop Color and Word Test: revised examiner’s manual*: Stoelting Co: Wood Dale, IL, 2002.
9. Kaplan E, Goodglass H, Weintraub S. *The Boston Naming Test.* Lea & Febiger: Philadelphia, 1983.
10. Strauss E, Sherman EMS, Spreen O. *A compendium of neuropsychological tests-third edition*. Oxford University Press: New York, 2006.
11. Matthews CG, Klove K. *Instructional manual for the Adult Neuropsychological Test Battery*. University of Wisconsin Medical School: Madison, WI, 1964.
12. Benton AL, Sivan A, Hamsher K, Varney N, Spreen O. *Contributions to neuropsychology assessment: a clinical manual.*Oxford University Press: New York, 1983.
13. Benton AL, Varney N, Hamsher K. Visuospatial judgment: a clinical test. Arch Neurol 1978; 35:364–367.
14. Hooper HE. *The Hooper Visual Organization Test manual.* Western Psychological Services: Beverly Hills, CA, 1958.
15. Wechsler D. *Wechsler Abbreviated Scale of Intelligence, Second Edition (WASI-II).* NCS Pearson: San Antonio, TX, 2011.
16. Delis DC, Kaplan E, Kramer JH. *Delis-Kaplan Executive Function System.* Psychological Corporation: San Antonio, TX, 2001.
17. Ruff RM. *Ruff Figural Fluency Test: professional manual*. Psychological Assessment Resources: Odessa, FL, 1996.
18. Bechara A. *Iowa Gambling Task: professional manual*. Psychological Assessment Resources: Lutz, FL, 2007.
19. Grace J, Malloy PF. *Frontal Systems Behavior Scale: professional manual.*Psychological Assessment Resources: Odessa, FL, 2001.
20. Fujita M, Hines CS, Zoghbi SS, Mallinger AG, Dickstein LP, Liow JS, et al. [Downregulation of brain phosphodiesterase type IV measured with 11C-(R)-rolipram positron emission tomography in major depressive disorder.](https://www-ncbi-nlm-nih-gov.ezproxyhost.library.tmc.edu/pubmed/22677471) Biol Psychiatry 2012;72(7):548-54.
21. [Yatham LN](https://www.ncbi.nlm.nih.gov/pubmed/?term=Yatham%20LN%5BAuthor%5D&cauthor=true&cauthor_uid=10868326), [Clark CC](https://www.ncbi.nlm.nih.gov/pubmed/?term=Clark%20CC%5BAuthor%5D&cauthor=true&cauthor_uid=10868326), [Zis AP](https://www.ncbi.nlm.nih.gov/pubmed/?term=Zis%20AP%5BAuthor%5D&cauthor=true&cauthor_uid=10868326). A preliminary study of the effects of electroconvulsive therapy on regional brain glucose metabolism in patients with major depression. J ECT 2000;16(2):171-6.
