# Supplementary material for: A longitudinal study on deep brain stimulation of the medial forebrain bundle for treatment-resistant depression
Source: Transl Psychiatry. 2018 Jun 4;8:111. doi: 10.1038/s41398-018-0160-4 (PMC5986795; doi:10.1038/s41398-018-0160-4)
Supplement: Supplementary file 2 — Supplemental table legend [file 41398_2018_160_MOESM2_ESM.docx]

**Supplementary Table Legend**

**Supplementary Table 1**: Neuropsychological Results at Baseline and 1 Year Follow-up with the Significance of the Mean Change. Note: M, mean Z score, SD, standard deviation, df = degrees of freedom; Ref. = Reference #; p values were corrected by FDR (false discovery rate). Ruff TUD, Ruff Figural Fluency Test-Total Unique Designs; Ruff TPE, Ruff Figural Fluency Test-Total Perseverative Errors; Ruff ER, Ruff Figural Fluency Test-Error Ratio; TMT A, Trail Making Test Part A; TMT B, Trail Making Test Part B; IGT, Iowa Gambling Task; Stroop, The Stroop Color and Word Test; FrSBe, Frontal Systems Behavior Scale (Self-Rating form); DKEFS Sorting Test CCS, Delis-Kaplan Executive Function System-Confirmed Correct Sorts; TOPF, Test of Premorbid Functioning; MoCA, Montreal Cognitive Assessment; CVLT-II LDFR, California Verbal Learning Test–Second Edition-Long Delay Free Recall; BLT LDFR, Brown Location Test-Long Delay Free Recall; BNT, Boston Naming Test; COWA, Controlled Oral Word Association Test; WASI-II, Wechsler Abbreviated Scale of Intelligence–Second Edition; WAIS-IV, Wechsler Adult Intelligence Scale–Fourth Edition; JLO, Judgment of Line Orientation Test; Hooper VOT, Hooper Visual Organization Test; GPT, Grooved Pegboard Test
